# Association of Acute-Phase IL-6 and SAA with Cardiovascular Events and Mortality Six Years After COVID-19 Infection: An Observational Cohort Study

**DOI:** 10.3390/ijms27114721

**Published:** 2026-05-24

**Authors:** Rumen Filev, Boris Bogov, Ralica Hadjieva, Krassimir Kalinov, Julieta Hristova, Dobrin Svinarov, Lionel Rostaing

**Affiliations:** 1 Department of Nephrology, Internal Disease Clinic, University Hospital “Saint Anna”, 1750 Sofia, Bulgaria; bbogov@yahoo.com (B.B.); rrhadjieva@gmail.com (R.H.); 2 Faculty of Medicine, Medical University Sofia, 1504 Sofia, Bulgaria; julieta_sd@yahoo.com (J.H.); dsvinarov@yahoo.com (D.S.); 3 Head Biometrics Group, Comac-Medical Ltd., 1404 Sofia, Bulgaria; krassimir.kalinov@comac-medical.com; 4 Department of Clinical Laboratory, University Hospital “Alexandrovska”, 1431 Sofia, Bulgaria; 5 Nephrology, Hemodialysis, Apheresis and Kidney Transplantation Department, Grenoble University Hospital, 38043 Grenoble, France; lrostaing@chu-grenoble.fr; 6 Internal Disease Department, Grenoble Alpes University, 38043 Grenoble, France

**Keywords:** COVID-19, SARS-CoV-2, interleukin-6, serum amyloid A, inflammation, cardiovascular risk, arrhythmia, myocardial infarction, mortality

## Abstract

Coronavirus disease 2019 (COVID-19) has been associated with an increased long-term cardiovascular risk, potentially mediated by magnitude of the acute inflammatory response inflammation. Interleukin-6 (IL-6) and serum amyloid A (SAA) are key components of the inflammatory cascade and may serve as biomarkers of post-COVID cardiovascular vulnerability. This longitudinal observational study investigated the association between post- COVID-19 infection IL-6 and SAA levels and major cardiovascular events over a six-year follow-up period. A total of 97 individuals with documented prior SARS-CoV-2 infection were included. Circulating IL-6 and SAA concentrations were measured in the acute phase. The composite endpoint included incident arrhythmia, myocardial infarction, and all-cause mortality. Biomarker distributions were right-skewed and were therefore analyzed using non-parametric methods and penalized logistic regression models. During follow-up, 14.4% of participants experienced the composite endpoint. Individuals with adverse outcomes had significantly higher IL-6 and SAA levels compared with event-free participants. IL-6 demonstrated the strongest association with mortality, whereas SAA showed particularly robust associations with the composite endpoint, and with myocardial infarction. Both biomarkers independently predicted long-term adverse events. Circulating IL-6 and SAA concentrations measured during the acute phase of SARS-CoV-2 infection were analyzed in relation to long-term cardiovascular outcomes. These findings support the hypothesis that the magnitude of the acute inflammatory response during SARS-CoV-2 infection may be associated with long-term cardiovascular outcomes and suggest that combined assessment of IL-6 and SAA may have potential utility for hypothesis-generating prognostic signal requiring validation, pending validation in larger studies.

## 1. Introduction

The coronavirus disease 2019 (COVID-19) pandemic, caused by the novel severe acute respiratory syndrome coronavirus 2 (SARS-CoV-2), represents the most significant global health crisis of the 21st century, resulting in millions of deaths and long-term health consequences worldwide [1]. Although early clinical attention focused primarily on acute respiratory failure, COVID-19 has increasingly been recognized as a systemic disease with substantial inflammatory and cardiovascular involvement [2,3].

A hallmark of severe COVID-19 is dysregulated immune activation characterized by excessive production of pro-inflammatory cytokines, commonly referred to as a “cytokine storm” [4]. Among these mediators, interleukin-6 (IL-6) plays a central role. IL-6 is a pleiotropic cytokine produced by immune cells, endothelial cells, and cardiomyocytes in response to infection and tissue injury. It regulates acute-phase protein synthesis, endothelial activation, coagulation pathways, and adaptive immune responses [5]. Elevated circulating IL-6 levels have been consistently associated with disease severity, respiratory failure, thrombotic complications, and mortality in patients with acute COVID-19 [3,4]. Importantly, IL-6 is also a well-established mediator of chronic low-grade inflammation, contributing to endothelial dysfunction, progression of atherosclerosis, plaque instability, arrhythmogenesis, and adverse cardiovascular remodelling [6,7]. It is therefore hypothesized that persistent elevation of IL-6 in the acute phase of SARS-CoV-2 infection may reflect inflammatory activity with long-term cardiovascular consequences.

Serum amyloid A (SAA) is another major acute-phase reactant predominantly synthesized by hepatocytes in response to stimulation by IL-6 and other pro-inflammatory cytokines. During acute inflammation, SAA concentrations increase markedly, often by several orders of magnitude [8]. Functionally, SAA participates in lipid metabolism, immune cell recruitment, modulation of high-density lipoprotein (HDL) structure and function, endothelial activation, and amplification of inflammatory cascades [8,9]. Notably, SAA has been implicated in atherogenesis, vascular inflammation, and thrombosis. Elevated SAA levels have been reported in severe COVID-19 and have been associated with adverse clinical outcomes [9]. Given its role at the interface between inflammation and vascular biology, SAA may represent a particularly relevant biomarker of long-term cardiovascular risk following SARS-CoV-2 infection.

Emerging epidemiological evidence indicates that individuals who survive SARS-CoV-2 infection remain at increased risk of a broad spectrum of cardiovascular complications, including arrhythmias, myocardial infarction, heart failure, thromboembolic events, and death, extending months to years beyond the acute phase [10]. Proposed mechanisms include persistent immune activation, endothelial dysfunction, microvascular injury, autonomic imbalance, and acceleration of pre-existing atherosclerotic disease. Increasing evidence supports chronic inflammation as a central unifying pathway linking SARS-CoV-2 infection to these long-term cardiovascular sequelae [7,10,11].

In this context, IL-6 and SAA are of particular interest as complementary biomarkers of post-COVID inflammatory burden. IL-6 reflects upstream cytokine-driven immune activation and systemic inflammation, whereas SAA represents a downstream acute-phase response closely associated with vascular and metabolic perturbations. Their combined assessment may therefore provide a more comprehensive characterization of magnitude of the acute inflammatory response inflammation and long-term cardiovascular vulnerability following COVID-19.

Beyond epidemiological associations, interventional evidence further supports a causal role of inflammation, particularly IL-6 signalling, in cardiovascular disease. The Canakinumab Anti-inflammatory Thrombosis Outcomes Study (CANTOS) demonstrated that targeting interleukin-1β significantly reduced recurrent cardiovascular events independently of lipid lowering, thereby confirming inflammation as a modifiable driver of atherosclerosis [12]. Subsequent analyses showed that reductions in IL-6 levels were strongly associated with improved cardiovascular outcomes, underscoring the pivotal role of IL-6 within the inflammatory cascade [10,13]. These findings provide mechanistic plausibility for the hypothesis that IL-6 elevation following SARS-CoV-2 infection may contribute to long-term cardiovascular risk.

Concomitantly, large epidemiological investigations have demonstrated that survivors of COVID-19 experience significantly increased risks of incident cardiovascular disease compared with non-infected controls, including among individuals who were not hospitalized during the acute phase [10]. The spectrum of excess risk encompasses ischemic heart disease, dysrhythmias, heart failure, thromboembolic events, and cardiovascular death [10]. Importantly, this elevated risk has been observed across a wide range of baseline cardiovascular risk profiles, suggesting that SARS-CoV-2 infection itself may act as an independent cardiovascular risk modifier.

From a mechanistic perspective, endothelial injury and immune activation associated with SARS-CoV-2 infection may initiate or accelerate key processes underlying atherothrombosis. Viral entry via angiotensin-converting enzyme 2 (ACE2) receptors expressed on endothelial and myocardial cells has been shown to contribute to endothelial dysfunction, microvascular inflammation, and a prothrombotic state (14). Persistent endothelial activation and immune dysregulation following clinical recovery may sustain vascular inflammation and promote plaque progression or destabilization. Furthermore, cardiovascular magnetic resonance imaging studies have documented myocardial involvement in recently recovered patients, demonstrating myocardial inflammation in a substantial proportion of individuals, irrespective of initial disease severity [11]. Chronic inflammatory signalling within the myocardium may contribute to electrical instability, structural remodelling, and increased susceptibility to arrhythmias. IL-6–mediated pathways have been implicated in the modulation of cardiac ion channel expression and myocardial fibrosis, providing a biologically plausible link between persistent cytokine elevation and long-term arrhythmic risk [14,15].

The present study aimed to evaluate the association between circulating IL-6 and SAA levels measured in the acute phase of SARS-CoV-2 infection and the occurrence of major adverse cardiovascular events and all-cause mortality over a six-year follow-up period. Specifically, this study sought to determine the long-term prognostic significance of these inflammatory biomarkers. By doing so, it aims to clarify the contribution of magnitude of the acute inflammatory response post-COVID inflammation to hypothesis-generating prognostic signal requiring validation and to identify potential biomarkers for the early identification of high-risk individuals who may benefit from intensified surveillance and preventive strategies.

## 2. Results and Discussion

### 2.1. Study Population and Follow-Up Outcomes

The final analysis included 97 individuals with a documented history of SARS-CoV-2 infection who were followed for six years after their index infection. The cohort exhibited a moderate baseline cardiovascular risk profile, characterized by a high prevalence of established risk factors, including hypertension, diabetes mellitus, and prior cardiovascular disease.

The inflammatory biomarkers IL-6 and SAA in acute phase of SARS-CoV-2 infection, demonstrated markedly right-skewed distributions, indicating substantial inter-individual variability and the presence of a subgroup with persistently elevated inflammatory activity (Table 1).

During the six-year follow-up period, 7 participants (7.2%) developed clinically significant arrhythmias, 9 participants (9.3%) experienced myocardial infarction, and 5 participants (5.2%) died. Overall, 14 participants (14.4%) experienced the composite endpoint (Table 2).

The observed event rates suggest a substantial long-term cardiovascular burden in this post-COVID cohort.

### 2.2. The Distribution of Inflammatory Biomarkers

Both IL-6 and SAA demonstrated significant deviations from normal distribution, as confirmed by formal statistical testing and visual inspection. The distributions were characterized by pronounced right skewness, with long right tails driven by a subset of participants exhibiting markedly elevated values. Consequently, all subsequent analyses of biomarker levels were conducted using non-parametric methods and are reported as medians with interquartile ranges.

Most participants exhibited relatively low IL-6 concentrations; however, a clinically meaningful minority (15.5%) demonstrated levels several-fold higher, suggesting the persistence of low-grade to moderate inflammation long after resolution of the acute infection. SAA showed even greater dispersion, consistent with its role as a highly sensitive acute-phase reactant that may remain elevated in chronic inflammatory states.

### 2.3. Association with Arrhythmia

Participants who developed arrhythmia during the follow-up period exhibited significantly higher post-COVID inflammatory biomarker levels compared with those who remained arrhythmia-free. The median IL-6 concentration in individuals who developed arrhythmia was approximately sixfold higher than in those without arrhythmia, and this difference was statistically significant according to the Mann–Whitney U test.

Similarly, SAA levels were significantly elevated in the arrhythmia group, with median values several-fold higher than in participants without arrhythmia. The magnitude and consistency of these differences indicate a strong association between both IL-6 and SAA levels and long-term arrhythmic risk following SARS-CoV-2 infection (Table 3).

Correlation analyses supported these findings, demonstrating moderate positive associations between both biomarkers and incident arrhythmia. These associations remained statistically significant after adjustment for major cardiovascular risk factors in ANCOVA model.

To account for potential confounders by baseline cardiovascular risk factors, adjusted analyses were performed using analysis of covariance (ANCOVA), including age, sex, obesity, hypertension, diabetes mellitus, and prior cardiovascular disease as covariates. Adjusted least-squares mean (LSMean) concentrations of IL-6 and serum amyloid A remained significantly higher among participants who subsequently developed arrhythmia, myocardial infarction, death, or the composite endpoint during follow-up (Table 3). The strongest adjusted association for IL-6 was observed for mortality, whereas SAA demonstrated particularly robust associations with myocardial infarction and the composite endpoint.

### 2.4. Association with Myocardial Infarction

**Figure 1:**
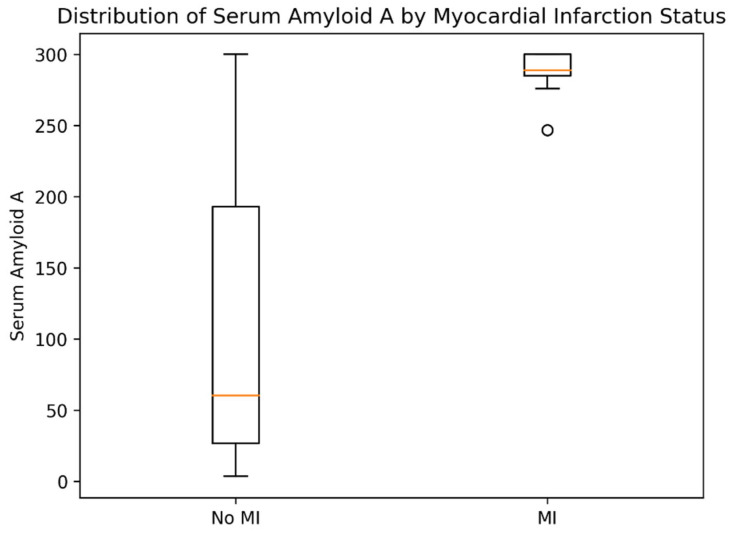
Distribution of Serum Amyloid A according to myocardial infarction status.

A similar but even more pronounced pattern was observed for myocardial infarction. Participants who developed myocardial infarction exhibited substantially elevated median IL-6 and SAA concentrations compared with those who did not experience infarction (Figure 1). Although IL-6 showed a statistically significant association, SAA demonstrated a particularly marked separation between the infarction and non-infarction groups, with minimal overlap between their respective distributions. Adjusted analyses confirmed these findings, demonstrating significantly higher adjusted LSMean concentrations of both IL-6 and SAA among participants who experienced myocardial infarction compared with those who did not.

These findings suggest that SAA may serve as a particularly sensitive marker of long-term ischemic risk in individuals recovering from COVID-19, potentially reflecting magnitude of the acute inflammatory response vascular or systemic inflammatory activity that promotes atherothrombotic events.

### 2.5. Association with Mortality

The most pronounced biomarker separation was observed for all-cause mortality. Participants who died during the follow-up period exhibited significantly higher IL-6 levels compared with survivors, with median values exceeding those of survivors by more than one order of magnitude. Although SAA levels were also significantly elevated among non-survivors, the contrast was particularly striking for IL-6.

Among all evaluated outcomes, IL-6 demonstrated the strongest association with mortality, suggesting it may reflect a dimension of systemic inflammatory burden linked to long-term outcomes. Adjusted ANCOVA analysis demonstrated that IL-6 remained strongly associated with mortality after controlling for age, sex, obesity, hypertension, diabetes mellitus, and prior cardiovascular disease (Table 3). These findings suggest that IL-6 may reflect a dimension of systemic inflammatory burden closely linked to long-term survival following SARS-CoV-2 infection.

### 2.6. Composite Endpoint Analysis

When arrhythmia, myocardial infarction, and death were combined into a composite endpoint, both IL-6 and SAA demonstrated strong associations. Participants who experienced an adverse event had significantly higher biomarker levels compared with those who remained event-free.

**Figure 2:**
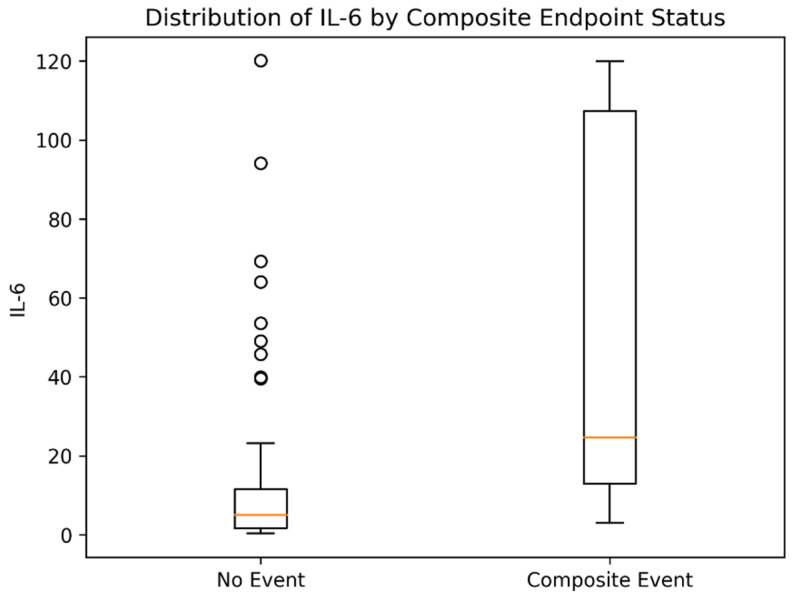
Distribution of IL-6 according to composite endpoint status.

Notably, SAA showed particularly robust discrimination, with markedly elevated median values in the composite-event group and comparatively lower concentrations among participants without events (Figure 2). Adjusted analyses demonstrated that both biomarkers remained significantly associated with the composite endpoint after accounting for baseline cardiovascular risk factors, with SAA exhibiting the strongest overall association (Table 3).

Collectively, these findings support the concept that magnitude of the acute inflammatory response post-COVID inflammation, as reflected by elevated IL-6 and SAA levels, is associated with a broad spectrum of adverse cardiovascular outcomes rather than a single isolated clinical phenotype.

### 2.7. Predictive Performance and Discrimination

For the composite endpoint, both biomarkers demonstrated strong predictive performance, with SAA exhibiting particularly high discriminatory capacity. Although the analyses demonstrated significant discrimination between participants with and without adverse outcomes, the present study was not powered to establish clinically applicable biomarker hypothesis-generating prognostic signal requiring validation. Therefore, the observed biomarker distributions should be interpreted as exploratory and hypothesis-generating.

Collectively, the findings underscore the distinct yet complementary roles of IL-6 and SAA in long-term risk prediction following recovery from SARS-CoV-2 infection. IL-6 demonstrated its strongest associations with mortality and arrhythmic outcomes, suggesting that it may reflect a systemic inflammatory burden closely linked to overall physiological resilience and long-term survival. In contrast, SAA showed particularly robust associations with myocardial infarction and the composite endpoint, supporting its potential role as a marker of magnitude of the acute inflammatory response vascular inflammation and atherothrombotic risk.

However, the limited number of events, particularly deaths, warrants cautious interpretation. Larger prospective studies are required to validate these findings, confirm their prognostic robustness, and clinically refine the applicable risk stratification.

The combined assessment of IL-6 and SAA therefore provides a more comprehensive characterization of long-term cardiovascular vulnerability following COVID-19 than either biomarker alone.

Given the observational nature of the present study, the identified associations between inflammatory biomarkers and long-term outcomes should not be interpreted as causal. Although the findings are biologically plausible and consistent with existing evidence linking inflammation to cardiovascular diseases, it remains unclear whether elevated IL-6 and serum amyloid A directly contribute to the development of adverse events or represent markers of underlying pathophysiological processes. Further studies, including larger prospective cohorts and interventional investigations targeting inflammatory pathways, are required to clarify causality.

This study has several limitations that should be considered when interpreting the findings. First, the sample size is relatively modest, with a limited number of outcome events, particularly for mortality, which may affect statistical power and the precision of effect estimates. However, this cohort represents a longitudinal analysis with a six-year follow-up after SARS-CoV-2 infection, a duration that remains rarely available, and required complete follow-up and biomarker data, inherently limiting cohort size.

Second, the observational design precludes causal inference, and the associations identified between inflammatory biomarkers and long-term cardiovascular outcomes should be interpreted as hypothesis-generating. Residual confounding cannot be excluded despite the collection of major cardiovascular risk factors, as unmeasured variables such as changes in medical therapy, lifestyle factors, lipid profiles, renal function trajectory, and COVID-19 disease severity or vaccination status may have influenced outcomes.

Third, IL-6 and SAA were measured at a single time point, in the acute phase and therefore do not capture longitudinal changes in inflammatory status. Serial biomarker measurements would be required to better characterize the persistence and dynamics of inflammation over time.

Fourth, the study cohort was derived from a single-centre population, which may limit the generalizability of the findings to other populations with different demographic characteristics, healthcare systems, or patterns of COVID-19 management.

Fifth, the composite endpoint included heterogeneous cardiovascular outcomes, which may reflect distinct underlying pathophysiological mechanisms. Although consistent associations were observed across endpoints, outcome-specific analyses should be interpreted with caution.

The absence of a non-COVID control group limits the ability to definitively attribute the observed long-term cardiovascular risk to SARS-CoV-2 infection itself, rather than to underlying baseline risk.

Maybe an important limitation of this study is the absence of serial clinical and laboratory assessments during the follow-up period. Inflammatory biomarkers were measured only during the acute phase of SARS-CoV-2 infection, and therefore do not reflect the longitudinal evolution of inflammatory status. Consequently, the study does not provide direct evidence of persistent inflammation, ongoing immune activation, or chronic inflammatory signaling following SARS-CoV-2 infection. Because participants with greater cardiovascular comorbidity may also exhibit higher inflammatory biomarker concentrations during acute illness, residual confounding by baseline cardiovascular vulnerability cannot be excluded.

Another important limitation is the presence of baseline cardiovascular comorbidity in a proportion of the cohort. Patients with pre-existing cardiovascular disease or cardiovascular risk factors may have been more vulnerable both to severe SARS-CoV-2 infection and to subsequent adverse outcomes, independently of COVID-19 itself. Therefore, elevated acute-phase IL-6 and serum amyloid A levels may reflect underlying cardiovascular vulnerability, systemic inflammatory burden, or reduced physiological reserve rather than a COVID-specific pathway leading to later cardiovascular events. Because of the observational design, modest sample size, and lack of a non-COVID control group, the present study cannot definitively separate the contribution of SARS-CoV-2 infection from pre-existing cardiovascular risk or intercurrent clinical events during follow-up. These findings should therefore be interpreted as hypothesis-generating and require confirmation in larger controlled studies.

In addition, participants may have experienced other medical conditions, infections, or changes in cardiovascular risk factors during the six-year follow-up period that were not systematically captured and could have influenced clinical outcomes. Therefore, the observed associations should be interpreted as reflecting the relationship between the magnitude of the acute inflammatory response to SARS-CoV-2 infection and long-term cardiovascular risk, rather than a continuous effect of inflammation.

However, the objective of the present study was not to assess persistent inflammation, but rather to evaluate whether the magnitude of the acute inflammatory response during SARS-CoV-2 infection is associated with long-term cardiovascular outcomes and mortality.

Despite these limitations, the study provides novel long-term data linking acute inflammatory biomarkers to cardiovascular outcomes over an extended follow-up period and supports the need for further large-scale, multicenter investigations.

## 3. Methods and Materials

### 3.1. Study Design and Population

This longitudinal observational cohort study was designed to evaluate the long-term association between inflammatory biomarkers measured during the acute phase of SARS-CoV-2 infection and subsequent cardiovascular outcomes and mortality. Participants with documented prior SARS-CoV-2 infection were enrolled following recovery from the acute phase of COVID-19. Infection was confirmed by reverse transcription polymerase chain reaction (RT-PCR) testing in accordance with internationally accepted diagnostic standards [16].

Patients were eligible for inclusion if they had laboratory-confirmed SARS-CoV-2 infection and available acute measurements of interleukin-6 (IL-6) and serum amyloid A (SAA). Individuals with active malignancy, systemic autoimmune disease, or documented acute inflammatory conditions at the time of biomarker assessment were excluded in order to minimize confounding related to non–COVID-associated inflammatory activity.

All participants provided written informed consent. The study protocol conformed to the ethical principles outlined in the Declaration of Helsinki and received approval from the institutional ethics committee [17,18,19].

### 3.2. Biomarker Assessment

Blood samples for IL-6 and SAA assessment were obtained during the acute phase of SARS-CoV-2 infection, as previously described in our earlier study evaluating IL-6 and SAA as predictors of short-term outcomes in COVID-19 patients with chronic kidney disease [20]. The present analysis extends that biomarker framework by examining whether inflammatory biomarker levels measured during the acute infection are associated with cardiovascular events and mortality during six years of follow-up.

Serum IL-6 concentrations were measured using a high-sensitivity enzyme-linked immunosorbent assay (ELISA) in accordance with the manufacturer’s specifications. The reference range for IL-6 was <7 pg/mL. Serum amyloid A concentrations were measured using the MAGLUMI 2000 (Snibe Co., Ltd., Shenzhen, China) fully automated chemiluminescence immunoassay (CLIA) platform, with a reference range of <10 mg/L. IL-6 was selected as a key upstream cytokine within the inflammatory cascade [5], whereas SAA was chosen as a major acute-phase reactant primarily induced by IL-6 signalling and closely linked to vascular inflammation and atherogenesis [8,9]. Both biomarkers have previously been associated with adverse cardiovascular outcomes in non-COVID populations [6,9].

### 3.3. Clinical Data and Outcomes

Baseline demographic characteristics, cardiovascular risk factors (including hypertension, diabetes mellitus, chronic kidney disease, and prior cardiovascular disease), and medication use were recorded at the time of enrolment. Cardiovascular risk factors were defined according to contemporary guideline criteria [19].

Participants were followed for six years after the index SARS-CoV-2 infection. The primary outcomes of interest were:

- Incident clinically significant arrhythmia,
- Myocardial infarction,
- All-cause mortality.

A composite endpoint was defined as the occurrence of any of the above events.

Myocardial infarction was diagnosed according to the Fourth Universal Definition of Myocardial Infarction, including clinical symptoms, elevation of cardiac biomarkers, and electrocardiographic or imaging findings consistent with myocardial ischemia [21]. Arrhythmias were defined as documented atrial or ventricular arrhythmias requiring medical therapy, hospitalization, or procedural intervention. Mortality data were obtained from hospital records and, when available, national registries.

### 3.4. Follow-Up Procedures

Clinical follow-up was conducted through scheduled outpatient visits and structured review of hospital records. For participants unable to attend in-person visits, structured telephone interviews and comprehensive review of electronic medical records were performed to ascertain clinical outcomes.

### 3.5. Statistical Analysis

Continuous variables were assessed for normality using the Shapiro–Wilk test in conjunction with visual inspection of histograms and Q–Q plots. As IL-6 and Serum Amyloid A (SAA) exhibited significant deviation from normal distribution, they were summarized as medians with interquartile ranges and compared between groups using the Mann–Whitney U test. Categorical variables were expressed as counts and percentages and compared using Fisher’s exact test when appropriate, given small cell sizes.

Associations between inflammatory biomarkers and clinical outcomes were evaluated using Spearman rank correlation coefficients. For predictive modeling, biomarker concentrations were log-transformed to reduce skewness and improve model stability. Penalized logistic regression was applied to estimate odds ratios, accounting for the limited number of outcome events and minimizing the risk of overfitting. Additionally, analysis of covariance (ANCOVA) models were constructed to evaluate adjusted differences in IL-6 and SAA concentrations according to clinical outcomes while controlling for age, sex, obesity, hypertension, diabetes mellitus, and prior cardiovascular disease. Adjusted least-squares means (LSMeans) with 95% confidence intervals were calculated. Optimal cutoff values were explored using the Youden index and are presented as hypothesis-generating.

All statistical tests were two-sided, and a *p*-value < 0.05 was considered statistically significant.

## 4. Conclusions

Six years after SARS-CoV-2 infection, elevated acute levels of inflammatory biomarkers in the acute phase were associated with adverse cardiovascular outcomes and mortality. Higher concentrations of IL-6 and serum amyloid A in the acute phase of the infection were associated with long-term cardiovascular outcomes as well as to a composite endpoint of major adverse—in this case 6 years later. IL-6 demonstrated the strongest association with mortality, whereas SAA exhibited particularly robust associations with myocardial infarction and the composite outcome, suggesting complementary biological and prognostic roles.

Measurement of IL-6 and SAA may facilitate the identification of high-risk individuals who could benefit from intensified surveillance and more aggressive cardiovascular risk factor modification.

Given the central role of IL-6 in orchestrating inflammatory responses and the vascular and metabolic effects of SAA, these biomarkers may capture complementary dimensions of persistent immune activation following SARS-CoV-2 infection. IL-6 may reflect upstream cytokine-driven systemic inflammatory burden, whereas SAA may serve as a sensitive indicator of vascular and atherothrombotic activity. Their combined evaluation may therefore enhance long-term risk prediction and provide mechanistic insight into post-COVID cardiovascular vulnerability.

## Figures and Tables

**Table 1:** Baseline characteristics of the study population (before COVID-19).

Variable	Value
Age, years	Median ~60 (IQR ~52–68)
Male sex	~50%
Hypertension	Common (>50%)
Diabetes mellitus	Frequent (31.4%)
Prior cardiovascular disease	Present in a substantial minority
Chronic kidney disease	Present in a subset
IL-6, pg/mL	Median ~5.6 (IQR ~3.2–11.4)
Serum Amyloid A, mg/L	Median ~65.0 (IQR ~40–120)

**Table 2:** Incidence of outcomes during six-year follow-up.

Outcome	n (%)
Arrhythmia	7 (7.2%)
Myocardial infarction	9 (9.3%)
Death	5 (5.2%)
Composite endpoint (any event)	14 (14.4%)

**Table 3:** Adjusted least-squares mean concentrations of IL-6 and serum amyloid A according to cardiovascular outcomes and mortality during six-year follow-up.

Least Squares Means *				
IL-6 <7 pg/mL	No Event	Event	Difference (95% CI Limits)	*p*-value
Arrythmia	14.794	56.571	−41.778 (−65.315; −18.241)	0.001
Myocardial Infarction	14.809	45.297	−30.488 (−52.858; −8.118)	0.008
Death	14.007	115.042	−101.035 (−122.461; −79.609)	<0.001
Composite	12.584	50.109	−37.525 (−54.700; −20.351)	<0.001
SAA <10 µg/mL				
Arrythmia	103.826	254.371	−150.545 (−231.395; −69.696)	<0.001
Myocardial Infarction	97.995	280.889	−182.893 (−253.505; −112.282)	<0.001
Death	108.123	251.119	−142.996 (−242.923; −43.068)	0.006
Composite	90.827	266.411	−175.583 (−229.452; 121.715)	<0.001

* Adjusted for age, sex, obesity, hypertension, diabetes mellitus, and prior cardiovascular disease using ANCOVA models.

## Data Availability

All of the data are available upon request from the corresponding author.

## Abbreviations

COVID-19	Coronavirus Disease of 2019
IL-6	Interleukin-6
SAA	Serum amyloid A
SARS-CoV-2	Severe acute respiratory syndrome coronavirus 2
SARS	Severe acute respiratory syndrome SARS
